# Simultaneous Generation of Surface Plasmon and Lossy Mode Resonances in the Same Planar Platform

**DOI:** 10.3390/s22041505

**Published:** 2022-02-15

**Authors:** O. Fuentes, I. Del Villar, I. Dominguez, J. M. Corres, I. R. Matías

**Affiliations:** 1 Electrical, Electronics and Communications Engineering Department, Public University of Navarra, 31006 Pamplona, Spain; omar.fuentes@unavarra.es (O.F.); ismel.dominguez@unavarra.es (I.D.); jmcorres@unavarra.es (J.M.C.); natxo@unavarra.es (I.R.M.); 2 Department of Telecommunications and Electronics, Pinar del Río University, Pinar del Río 20100, Cuba; 3 Institute of Smart Cities (ISC), Public University of Navarra, 31006 Pamplona, Spain

**Keywords:** thin-films, sensors, surface plasmon resonance, lossy mode resonance, planar waveguide

## Abstract

A planar waveguide consisting of a coverslip for a microscope glass slide was deposited in one of its two faces with two materials: silver and indium tin oxide (ITO). The incidence of light by the edge of the coverslip permitted the generation of both surface plasmon and lossy mode resonances (SPRs and LMRs) in the same transmission spectrum with a single optical source and detector. This proves the ability of this optical platform to be used as a benchmark for comparing different optical phenomena generated by both metal and dielectric materials, which can be used to progress in the assessment of different sensing technologies. Here the SPR and the LMR were compared in terms of sensitivity to refractive index and figure of merit (FoM), at the same time it was demonstrated that both resonances can operate independently when silver and ITO coated regions are surrounded by different refractive index liquids. The results were supported with numerical results that confirm the experimental ones.

## 1. Introduction

Surface plasmon resonances (SPRs) and lossy mode resonances (LMRs) have been widely explored in the literature [1,2,3,4,5,6]. Both are generated under the presence of a thin film, and their utilization as a sensor depends on the thickness of the thin film, its refractive index, its extinction coefficient, the refractive index of the waveguide and the surrounding medium [7].

As for the differences, there are several that exist between these two types of resonances. For the SPRs, the range of materials that can be used is limited to the metals (gold, silver, aluminum, copper). In the case of LMRs, there is a much wider variety of materials that can meet the conditions for their generation [8], such as conductive oxides [3], polymeric films [6] or polymer/metal oxide hybrids [9]. The price of these materials is relatively cheaper and access to these materials is also easier. Furthermore, its use is beneficial for the mass production of sensors based on LMRs.

The difference in the materials required for the generation of each resonance can be explained by the relation between the permittivity of the thin-film and the media surrounding it. SPRs are obtained when the real part of the thin-film permittivity is negative and higher in magnitude than both its own imaginary part and the permittivity of the material surrounding the thin film, whereas LMRs occur when the real part of the thin-film permittivity is positive and higher in magnitude than both its own imaginary part and the material surrounding the thin film [10]. As an example, Table 1 shows the conditions under the assumption that the surrounding medium refractive index is 1 (air).

Another different aspect is the range of incidence angles suitable for the generation of resonances. SPRs are optimally obtained for angles ranging between 40° and 70° [8,11], whilst LMRs generally occur for an angle close to grazing incidence, that is, an angle that approaches 90° [12]. This explains why most of the experimental work in LMRs uses fiber optics instead of the Kretschmann–Raether configuration [6,13,14,15,16], since, with this last configuration, it is very difficult to impinge the light at 90°.

**Figure 1:**
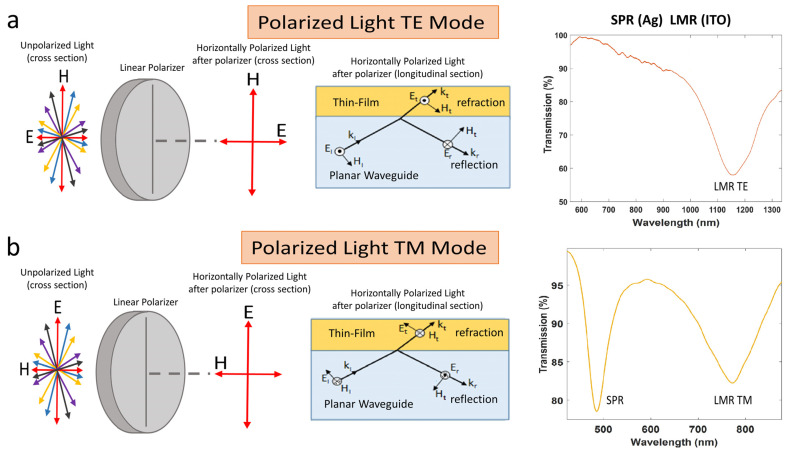
Schematic view of SPR and LMR excitation for: (**a**) Horizontally polarized light (only LMR_TE_ is excited); (**b**) Vertically polarized light (SPR and LMR_TM_ are excited).

On the other hand, SPRs can only be excited with transversal magnetic (TM) polarized light, while LMRs can be excited with both: transversal electrical (TE) and transversal magnetic (TM) polarized light [6]. Furthermore, although only a light source and an optical spectrum analyzer are required to perform experiments on both phenomena, the control of the polarization greatly improves resolution. In Figure 1, a schematic is shown for the excitation of a thin-film coated waveguide with TE or TM polarized light. For TM polarized light, both LMRs and SPRs can be generated, whereas for TE polarized light only LMRs are generated, as will be demonstrated later.

Another difference between LMRs and SPRs is that in LMRs the position of the resonance generated by an LMR in the optical spectrum depends directly on the thickness of the coating, which allows a simple adjustment of the wavelength [17], whilst it is not so easy to establish this relationship in the case of the SPR.

Also from the spectral point of view, the differences between SPR and LMR are more notable: SPRs present a resonance that disappears when the thickness of the coating reaches a certain value [18], whilst with LMRs, multiple resonances can be generated (LMR_1_, LMR_2_, LMR_3_, etc.) in a wide range of the spectrum (VIS/NIR). This characteristic can be obtained simply by varying the thickness of the coating and without the need to modify the geometry of the waveguide. This property makes LMRs suitable for manufacturing multi-peak and multi-variable sensors or for designing multi-wavelength optical filters [6]. Moreover, it has been demonstrated that materials with very different properties depending on the operating wavelength, such as indium tin oxide (ITO), can be used for generating LMRs in the visible region and SPRs in the near infrared (NIR) with a Kretschmann–Raether configuration and compare their properties [6,13].

Here, it will be demonstrated that it is not necessary to use materials with special properties to generate both SPRs and LMRs. In addition, a simpler configuration than Kretschmann–Raether will be used, which permits us to visualize better LMRs, something very difficult with the Kretschmann–Raether configuration since angles approaching 90° are required. The configuration consists of launching light on the edge of a coverslip for microscope glass slide, something successfully used for generation of LMRs by coating one face of the coverslip [19]. Moreover, it was recently demonstrated the ability to generate two independent LMRs in the same transmission spectrum by coating both faces of the coverslip [20], suggesting the possibility to generate a SPR and a LMR by deposition of a metallic thin film and a dielectric thin film. This is what will be done here: a silver and an ITO coating will be deposited on the same substrate to generate an SPR and an LMR with a single light source and spectrometer. This will demonstrate that this setup can be used for analyzing and comparing different optical phenomena, concretely here for comparing the sensitivity and figure of merit (FoM) of an SPR and an LMR in the visible region. Clearly, this opens the possibility to use each part of the structure for detecting different biological or chemical species, depending on the properties of each of the materials deposited, which can be selectively functionalized, be deposited with a second sensitive coating, etc.

## 2. Materials and Methods

### 2.1. Experimental Setup

**Figure 2:**
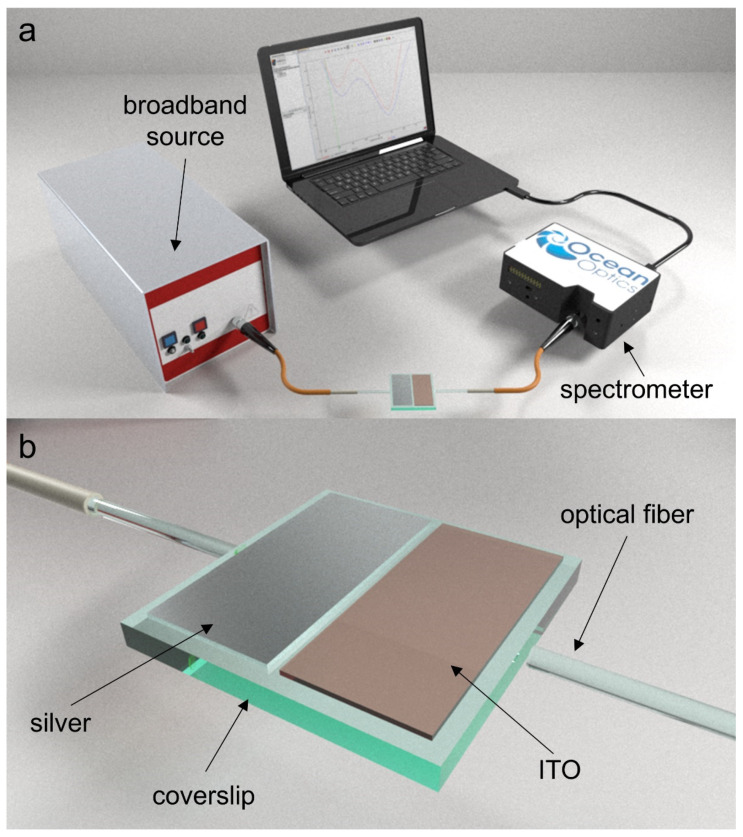
(**a**) Experimental setup. (**b**) Coverslip nanocoated with silver and ITO nanofilms.

One face of the coverslip was deposited, half of the surface with silver and another half of the surface with indium tin oxide (ITO). For this purpose, a DC sputtering machine (K675XD from Quorum Technologies, Ltd.) was used. The method used to generate the rectangular patterns was a two-step process based on the utilization of a mask with a hole. In the first step the mask was placed on top of the coverslip to generate the silver coating with a silver target in the sputtering machine, and in the second step the mask was shifted to generate the ITO coating with an ITO target in the sputtering machine. The ITO sputtering target (57 mm in diameter and 3 mm in thickness) was purchased from ZhongNuo Advanced Material Technology Co and the silver sputtering target from Plasmaterials (57 mm in diameter and 1 mm in thickness). The parameters of the DC sputtering machine used in the experiment were an argon partial pressure of 7 × 10^−2^ mbar and an intensity 140 mA for the ITO coating, whereas for the silver coating an argon partial pressure of 7 × 10^−2^ mbar and an intensity 35 mA was used. The set-up used is very simple and it is presented in Figure 2a. 

It consists of a halogen broadband source DH-2000 and a USB2000 spectrometer, both from OceanOptics Inc., Largo, FL, USA. Figure 2b shows the schematic of the coverslip coated with silver and ITO, including a linear polariser LPVIS050 from Thorlabs between the output of the optical fiber that launched light in the waveguide and the waveguide itself. This permits the excitation of the waveguide with linearly polarized light, which can be oriented horizontally or vertically (i.e., the electric field is oriented horizontally or vertically). This allows for the separation of LMR_TE_ or LMR_TM_, which are deeper and easier to track when the device is used as a sensor.

### 2.2. Thin Film Characterization

**Figure 3:**
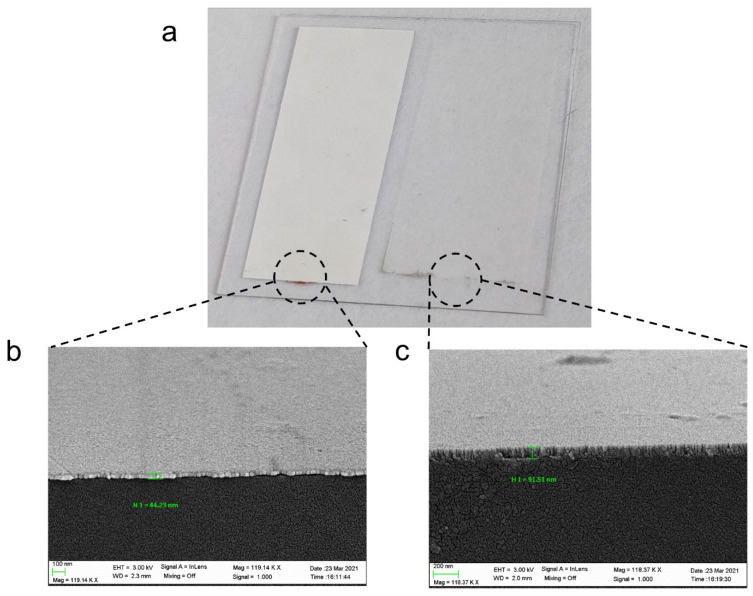
(**a**) Coverslip deposited with silver (left part) and ITO (right part). The separation between both thin films is 1 mm. (**b**) FESEM image of the cross section of the part of the coverslip deposited with silver. (**c**) FESEM image of the cross section of the part of the coverslip deposited with ITO.

Figure 3 shows the thin films of silver and ITO deposited on a coverslip, characterized with a field emission scanning electron microscope (model UltraPlus FESEM from Carl Zeiss Inc., Thornwood, NY, USA) with an in-lens detector at 3 kV and an aperture diameter of 30 μm). The thickness of both coatings was selected in such a way that both SPRs and LMRs will be seen without overlapping in the Results section. The ITO thickness is adequate for seeing the LMR_TM_ and short wavelengths and the LMR_TE_ at long wavelength in air and in liquid. Regarding the silver coating, in order to avoid overlapping with the LMR, the deposited coating will permit us to see the SPR in liquid at short wavelength, far from the other two resonances in liquid located at longer wavelengths. In addition, the gap between the silver and the ITO region was 1 mm. The utilization gap is only for the sake of an easier characterization of the refractive index sensitivity in the results section, since the separation permits the pouring of the liquid alternatively in both regions. However, the gap plays no role in the results, something that has been verified both theoretically and experimentally.

## 3. Results

**Figure 4:**
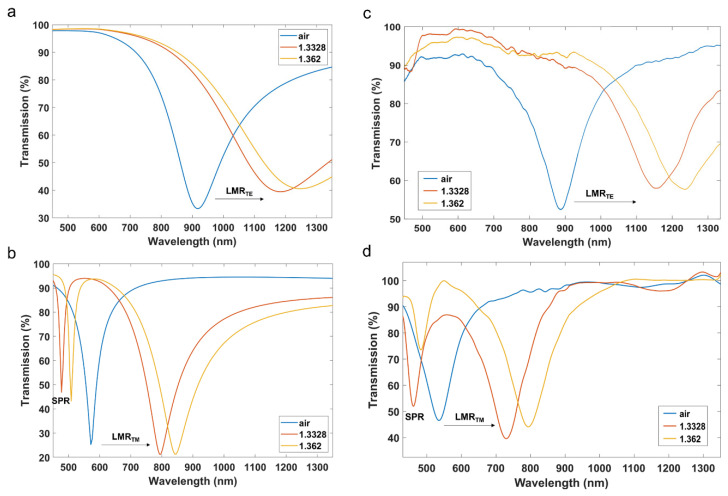
Transmission spectra for three different surrounding medium refractive indices (1, 1.3328 and 1.362): Simulation results at (**a**) TE polarization and (**b**) TM polarization; experimental at (**c**) TE and (**d**) TM polarization.

On the basis of the thickness of silver and ITO, and the ellipsometric models of silver and ITO [21,22], the transmission spectrum was calculated by means of a numerical analysis based on the well-known plane wave method for a one-dimensional multilayer waveguide [23], validated for both SPRs [24] and LMRs [17]. Figure 4a,b show the numerical results at both TE and TM polarization for different surrounding medium refractive indices: air, water and ethanol/water (70:30, *v*/*v*), which were measured with a refractometer 30GS from Mettler Toledo Inc. This device obtains the refractive index for a wavelength of 589.3 nm and the calculated refractive index was, respectively, 1, 1.3328 and 1.362.

Figure 4c,d show the corresponding experimental data, which agree with the simulation data. This certifies that thin film is homogeneous, because the thin film thickness calculated by the microscope, introduced in the simulation tool, leads to the same position of the SPR/LMRs in Figure 4a,b.

Another conclusion that can be extracted from the numerical and experimental results is that an SPR resonance corresponding to the silver thin film is observed at around 500 nm only for TM polarization, whereas the LMR generated by the ITO thin film can be visualized at longer wavelengths at both TE and TM polarization. This is one of the main differences between SPRs and LMRs [3,6].

**Figure 5:**
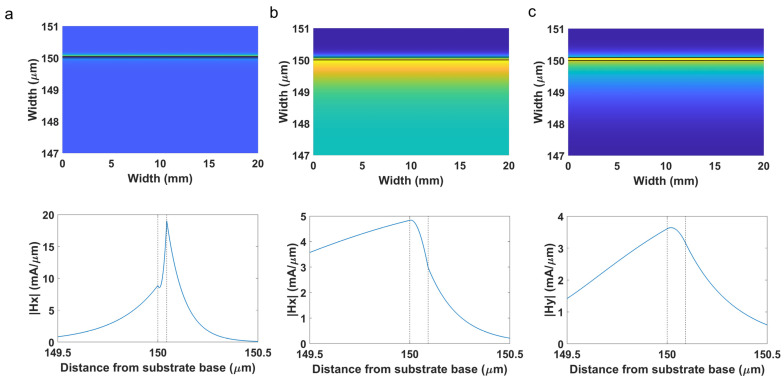
Optical field intensity distribution and transversal magnetic field in the upper part of the cross section of the 150 µm coverslip coated with (**a**) silver (mode TM_1_ at wavelength 450 nm) and (**b**,**c**) ITO (modes TE_0_ and TM_0_ at wavelengths 1050 and 750 nm respectively).

The simulation also permits us to analyze the optical field intensity. This will allow us to confirm that the resonance at shorter wavelengths is an SPR, and the resonance at longer wavelengths is an LMR. To this end, the optical field intensity and the transversal magnetic field of modes TM_1_ in the silver coated region and TE_0_ and TM_0_ in the ITO coated region were calculated with FIMMPROP, an integrated module of FIMMWAVE, for a SMRI 1.3328 (water) at wavelengths 450, 700 and 1050 nm, respectively (see Figure 5). These wavelengths were selected because they are in the proximity of the central wavelength of the SPR (480 nm in Figure 4b) the LMR_TM_ (795 nm in Figure 4b) and the LMR_TE_ (1185 nm in Figure 4a). The reason for the selection of wavelengths shifted to the blue related to the central resonance wavelength was that it is easier to visualize the confinement of the mode that is responsible for the generation of the resonance. 

The finite difference method (FDM) with the Quasi 2D version was used to calculate the modes and the fields in the cross section of the waveguide. Regarding the refractive index of the waveguide, since both the microscope slides and the coverslips were made of soda lime glass, the refractive index model of [25] was used. The planar waveguide was placed on a poly(methyl methacrylate) (PMMA) substrate material and the refractive index of this material was modelled according to [26].

The main conclusion that can be extracted from Figure 5 is that the SPR presents an evanescent field that decays faster than the LMR_TE_ and the LMR_TM_. This will have implications in terms of a higher sensitivity in the proximities of the thin-film in the case of the silver coated region, whereas the ITO coated region will be sensitive in a longer range but not so much in the proximities of the thin film.

**Figure 6:**
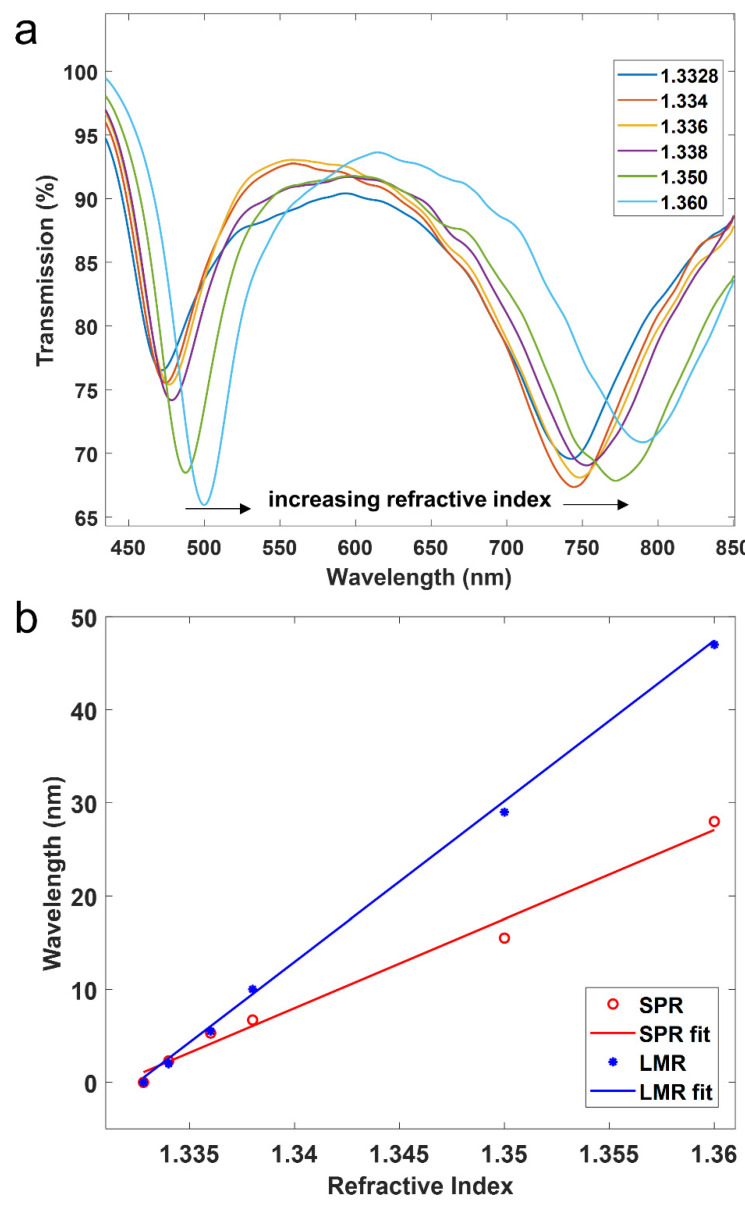
(**a**) Transmission spectra (TM polarization) for different surrounding medium refractive indices. (**b**) Relative wavelength shift as a function of refractive index for both the LMR and SPR.

In order to analyze the performance of the device as a sensor, the two regions of the coverslip were covered with liquids of different refractive indices (water and ethanol 70%) in the range from 1.3328 to 1.3620. For the sake of simplicity, only the case of TM polarization was analyzed because it permits us to visualize in the same spectrum the SPR and the LMR_TM_ (see Figure 6). The results show that both resonances experience a wavelength shift to the red as a function of the refractive index, though the sensitivity for the LMR (1720.6 nm/RIU) is higher than the sensitivity of the SPR (1029.4 nm/RIU). However, it must be pointed out that the sensitivity in optical sensors typically increases at longer wavelengths. Therefore, no big difference in terms of sensitivity can be extracted from these results [22]. In addition, these sensitivity values are in the typical range of sensitivity observed with other materials deposited on coverslips and characterized with the same setup used here [19,22], and when compared to other optical sensors, they are in a good position in the global scale, though sensors do exist with one order of magnitude higher. Generating the resonance in the near infrared could improve the sensitivity [27].

Regarding the figure of merit (FoM), this parameter is defined as the sensitivity divided by the full-width at half maximum (FWHM). In Figure 4b, the depth of the resonance is around 50%. Therefore, in order to solve this issue and obtain a good comparison, here we will calculate this parameter as 75% of the maximum. In that case, by extracting the FWHM of the red curve in Figure 4b, FoM is 20.59 for the SPR and 10.52 for the LMR. This better performance of the SPR can be explained by the lower FWHM.

**Figure 7:**
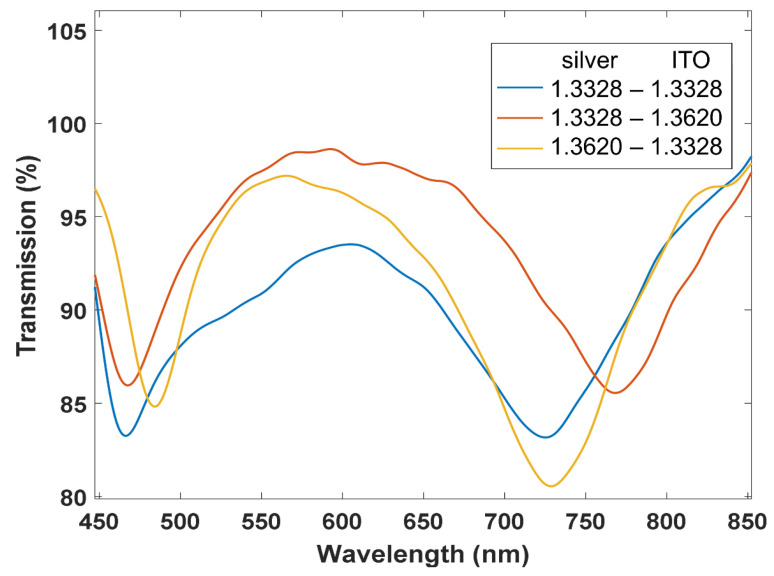
Transmission spectra (TM polarization) when both the silver and the ITO coated are covered by the same refractive index (1.3328-1.3328, blue line), when the silver coated region is immersed in refractive index 1.3328 and the ITO coated region in 1.362 (red line), and when the silver coated region is immersed in refractive index 1.362 and the ITO coated region in 1.3328 (yellow line).

In addition, in Figure 7 some results are shown on the effect of covering the silver and the ITO coated regions with different liquids. The results indicate that both resonances operate independently and, therefore, the sensitivity in each peak is the same as in a setup where either of the coatings is deposited separately. 

Regarding the depth of the LMRs, some changes can be observed in Figure 7. Here, it must be pointed out that LMRs with incidence of light by the edge of the coverslip are very sensitive in terms of depth of the resonance when the coverslip is manipulated. The setup is not automated and there is a need to remove the coverslip from the setup every time new liquids are set on top of the coverslip. This explains inaccuracies in terms of depth of the resonance, which is affected by changes in the coupling of light to the coverslip. However, this is not the case with the wavelength, which is a more reliable measurement. The problem of the depth could be solved with an automated system.

## 4. Conclusions

To conclude, it has been demonstrated that a single coverslip for a microscope glass slide can be excited by the lateral edge with a broadband source to generate a transmission spectrum where an SPR and an LMR resonance can be generated simultaneously. This permits the evaluation of both phenomena at the same time in terms of refractive index and opens the field to the generation of multiresonance spectra where different materials can be explored, since SPRs and LMRs are generated by complementary materials: metallic and dielectric materials, respectively. Moreover, this platform could be used for analyzing other phenomena such as localized surface plasmon resonance (LSPR), comparing nanopatterns more complex than a uniform thin-film, or analyzing the effect of a biofilm or a gas on different resonance supporting structures in a simple benchmark platform like the one proposed in this work. Regarding the applicability of the device in terms of sensing, the structure presented here has been explored for a wavelength range from 400 to 1000 nm. However, the sensitivity could be increased by operating at longer wavelengths. Furthermore, a broad range of wavelengths could be explored in the single system, which could allow us to obtain a higher number of resonances in the same spectrum, each one generated by coatings of different sensitivity, those with higher sensitivity for applications with a short refractive index range and those with lower sensitivity for applications with a high refractive index range, converting the system in a multipurpose platform.

## Figures and Tables

**Table 1:** Conditions for the generation of SPRs and LMRs (*ε*_1_, *n*_1_, and *k*_1_ represent the permittivity, refractive index and extinction coefficient for the substrate, whilst *ε*_2_, *n*_2_, and *k*_2_ represent the permittivity, refractive index and extinction coefficient for the thin film).

Type of Resonance	$\hat{\varepsilon }$ Conditions	n, k Conditions
Surface Plasmon Resonances (SPR)	$\varepsilon_{2}^{\prime }<0$ |$\varepsilon_{2}^{\prime }|$>|$\varepsilon_{2}^{\prime \prime }|$ |$\varepsilon_{2}^{\prime }|$>|$\varepsilon_{1}^{\prime }|$	$\begin{array}{c} k_{2}>\left(\sqrt{2}+1\right)n_{2}\\ k_{2}^{2}-n_{2}^{2}>n_{1}^{2} \end{array}$
Lossy Mode Resonances (LMR)	$\varepsilon_{2}^{\prime }>0$ |$\varepsilon_{2}^{\prime }|$>|$\varepsilon_{2}^{\prime \prime }|$ |$\varepsilon_{2}^{\prime }|$>|$\varepsilon_{1}^{\prime }|$	$\begin{array}{c} k_{2}<(\sqrt{2}-1)n_{2}\\ n_{2}^{2}-k_{2}^{2}>n_{1}^{2} \end{array}$

## Data Availability

The data that support the findings of this study are available from the corresponding authors upon request.
